# Adjunctive rifampin therapy for diabetic foot osteomyelitis: A protocol for systematic review and meta-analysis

**DOI:** 10.1097/MD.0000000000020375

**Published:** 2020-05-29

**Authors:** Yanli Zhang, Shengju Wang, Min Liu, Shasha Yao, Song Fang, Haiping Cheng, Qiu Chen

**Affiliations:** Department of Endocrinology, Hospital of Chengdu University of Traditional Chinese Medicine, Chengdu, P.R. China.

**Keywords:** diabetic foot, osteomyelitis, protocol, rifampin, systematic review and meta-analysis

## Abstract

**Introduction:**

The prevalence of diabetes continues to rise around the world. Diabetic foot is a serious complication of diabetes, and diabetic patients with diabetic foot osteomyelitis (DFO) have a fourfold increased risk of amputation, usually indicating death. Therefore, it is particularly important to seek a more effective treatment for DFO. The treatment of DFO varies from person to person, and antimicrobial therapies vary widely. A large number of clinical studies have shown that rifampicin adjuvant therapy can reduce the rate of amputation and mortality in DFO patients. However, there is no systematic summary of clinical evidence, which limits the clinical application of rifampicin. Therefore, we attempted to provide high-quality evidence for the clinical efficacy and safety of rifampin in the adjuvant treatment of DFO through this meta-analysis.

**Methods:**

English literature is mainly searched in Cochrane Library, PubMed, EMBASE and Web of Science, while Chinese literature is from CNKI, CBM, VIP and Wangfang databases. At the same time, we will search clinical registration tests and gray literature. Two methodologically trained researchers will read the title, abstract, and full text, and independently select qualified literature based on inclusion and exclusion criteria. Binary data is expressed as relative risk, continuous data is expressed as mean difference or standard mean difference. The final data are synthesized using a fixed effect model or a random effect model, depending on the presence of heterogeneity. In the end, the patient's amputation rate and mortality were the main research indicators. Survival rate, HbA1c, serum creatinine, changes in ulcer area, and SF-36 quality of life assessment were used as secondary indicators. We will perform a sensitivity analysis to assess the stability of the results. Then the publication bias was evaluated by funnel plot analysis and Egger test. Finally, we will use a “recommendation grading, evaluation, formulation and evaluation” system to assess the quality of the evidence. All data analysis will be meta-analyzed by the statistical software RevMan software version 5.3.

**Results:**

This study will provide a high-quality comprehensive report on the effectiveness and safety of rifampicin in the treatment of DFO, and our findings will be published in peer-reviewed journals.

**Conclusion:**

This systematic review and meta-analysis will provide a comprehensive summary and careful evaluation of rifampicin as an adjuvant treatment of DFO with a view to providing multiple options for clinical treatment of the disease.

**Registration number:**

is INPLASY202040084.

## 1 Introduction

Diabetes mellitus is a metabolic disorder characterized mainly by the presence of chronic hyperglycemia due to a deficiency of insulin secretion or insulin resistance.^[1]^ The International Diabetes Federation reported 425 million people globally with diabetes mellitus in 2017.^[2]^ These figures were expected to increase to 693 million by 2045.^[3]^ Long-term hyperglycemia can lead to a series of serious complications, such as diabetic nephropathy, diabetic peripheral neuropathy, diabetic retinopathy and diabetic foot, diabetic foot is considered to be 1 of the most serious complications. It is 1 of the main causes of disability and death of diabetic patients, having the characteristics of great difficulty in treatment, long period, high cost and high disability and fatality rate, reduces the quality of life of patients, has become a serious burden of modern society.^[4–6]^ Amputation is a more serious consequence of diabetic foot than death. About 15% of diabetic patients will eventually develop foot ulcers, and infection is 1 of the main reasons to accelerate the occurrence and development of diabetic foot. According to the severity and chronicity of infection, 20% to 60% of diabetic foot infections are related to bones. diabetic foot osteomyelitis (DFO) occurs when diabetic foot infections invade bones, and the risk of amputation will increase by 4 times.^[7]^ Therefore, the control of bone infection in diabetic foot patients is an important measure to improve the limb salvage effect of diabetic patients.

Antimicrobial therapy for diabetic foot infections varies widely. The treatment methods for DFO are also different, with different effects. Adjunctive rifampin therapy is commonly employed in Europe,^[8]^ where 56 to 100% of practitioners select oral antimicrobial therapy with adjunctive rifampin for osteomyelitis including DFO.^[9–11]^

Rifampin has unique antimicrobial properties that make it a useful adjunctive therapy for osteomyelitis. It penetrates osteoblasts and retains antimicrobial activity within these cells.^[12]^ Rifampin also penetrates biofilms and retains activity within them.^[13]^ When antimicrobial therapies are withdrawn, the persister cells may reactivate to cause recurrent infections. Rifampin also demonstrates potent activity against persister cells in biofilms that exceeds that of any other currently available antibiotic.^[14]^ Adjunctive rifampin therapy has improved outcomes in several studies of osteomyelitis outside the setting of the diabetic foot.^[15,16]^ Rifampin has broad spectrum activity against gram-positive organisms, which are the most common pathogens in DFO. S. aureus is the most common bacteria recovered from bone cultures in DFO. Other gram-positive organisms, including coagulase negative staphylococci and streptococci are recovered from 30% to 70% of cases.^[17]^ Gram-negative organisms are found in a minority of cases of DFO. Clinical activity of rifampin against gram-negative pathogens has been observed in combination therapy of serious gram-negative infections that had failed other therapies.^[18,19]^ Antimicrobial activity from rifampin may consequently be seen in most cases of DFO.

In retrospective and prospective randomized clinical trials, the addition of rifampin has reduced relapse rates from chronic osteomyelitis, improving arrest of osteomyelitis by 28% to 42% compared with regimens without rifampin.^[15,20,21]^ There have been several studies showing that patients treated with rifampicin have lower amputation rates and mortality compared with patients who have not received rifampicin. These existing clinical trials have shown that adding Rifampicin may be an effective antibacterial drug for DFO.^[9,22,23]^

However, the evaluation of the effectiveness of rifampicin in the treatment of DFO only by a single clinical study is sometimes more 1-sided, while the systematic review is more comprehensive and less biased. It can comprehensively retrieve previous related studies and strictly evaluate its quality and the conclusions are more reliable than single clinical studies. At present, there is no systematic review evaluation to explore the efficacy of rifampin in the adjuvant treatment of DFO. Therefore, we intend to collect clinical randomized controlled trials of rifampicin adjuvant therapy in the DFO on the basis of evidence-based medicine, and conduct a systematic review and meta-analysis of its efficacy and safety to provide better clinical evidence for rifampin adjuvant therapy DFO.

## 2 Methods

### 2.1 Protocol registration

The systematic review and meta-analysis protocol has been registered on the INPLASY website (https://inplasy.com/inplasy-2020-4-0084/) and INPLASY registration number is INPLASY202040084. The protocol was reported according to the Cochrane Intervention Systematic Review Manual and guidelines for the preferred reporting project of the Systematic Review and Meta-Analysis Protocol (PRISM).^[24]^ If there are any adjustments in the whole study, we will report and refine the details in the final report.

### 2.2 Inclusion criteria

#### 2.2.1 Study design

This study only selected clinical randomized controlled studies of rifampicin for the adjuvant therapy of DFO. Animal experiments, observational studies, cohort studies, reviews, case reports, and non-randomized controlled trials were excluded.

#### 2.2.2 Participants

Regardless of age, gender, ethnicity, and other factors, patients who met the DFO diagnostic criteria of the International Diabetes Working Group were included.^[25]^ Exclude patients who are using the drug with contraindications to rifampicin and patients who are allergic or intolerant to rifampicin.

#### 2.2.3 Interventions

Both groups of patients received the routine diabetes treatment recommended by the ADA guidelines, including diet, exercise, hypoglycemic, lipid-lowering and other basic treatments of diabetic foot.^[26]^ The test group was given rifampicin, and the control group was given other antibiotics, placebo or no drugs. In addition, neither group of patients can take drugs that interfere with the outcome indicators, and the treatment time is ≥6 weeks.

### 2.3 Outcomes

#### 2.3.1 Primary outcomes

The primary outcomes of this study include evaluating the difference between the efficacy of adjuvant treatment for DFO with and without rifampin, That is to evaluate the effect of rifampicin in the treatment of DFO on amputation rate and mortality in patients with diabetic foot.

#### 2.3.2 Secondary outcomes

The secondary outcomes mainly included the survival rate of unamputeed patients, changes in HbA1c, serum creatinine, ulcer area, and assessment of patient quality of life by SF-36.

### 2.4 Study selection

**Figure 1:**
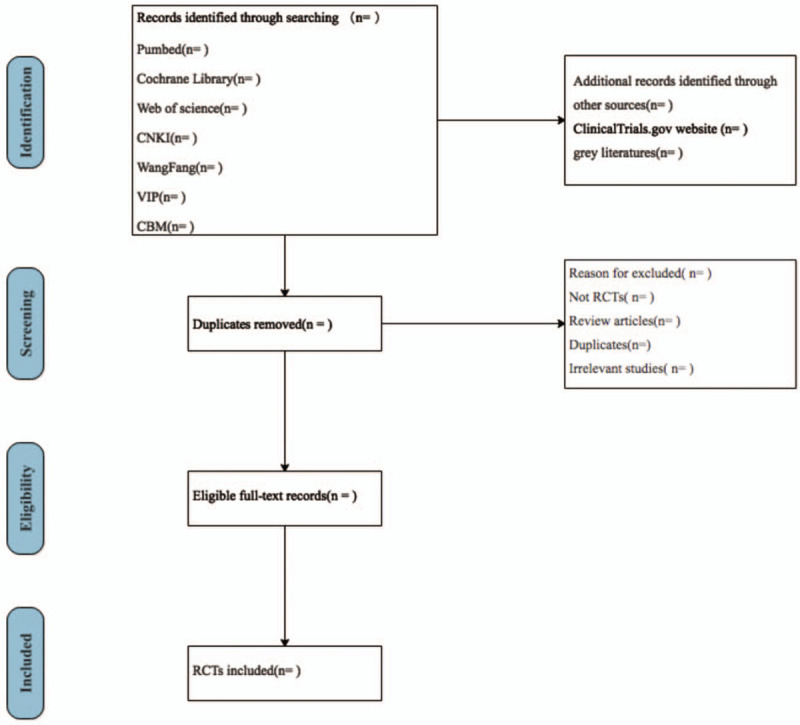
Flow chart of the study selection.

Two reviewers independently searched all the literatures, and literatures related to this study will be imported into the EndNote software. After that, delete duplicate records. Reviewers first screened the titles and abstracts of each citation to identify potentially eligible studies, and then reviewed the full text to determine whether it was included. If there is any objection, it should be negotiated or resolved with the third reviewer. Our findings will be reported using the recommended methodology and the list of preferred reporting items in the systematic review and meta-analysis.^[24]^Figure 1. describes the process of identifying and selecting studies.

### 2.5 Study search

We will retrieve each database from the built-in until June 2020. The English databases we searched are PubMed, Embase, Cochrane Library Central Register of Controlled Trials and Web of Science; Chinese databases include China National Knowledge Infrastructure (CNKI) database, Wanfang Data Knowledge Service Platform, the VIP information resource integration service platform (cqvip), China Biology Medicine Disc (Sino Med) with a language limitation of English and Chinese. In addition, we searched Google Scholar, Baidu Scholar, and unpublished research and other related literature, most importantly, we manually searched the Chinese Clinical Trial Registry (ChiCTR) and ClinicalTrials.gov related research in the Chengdu University of Traditional Chinese Medicine Library. We use keywords and free words combination as the basic search strategy. The search criteria are as follows: “Diabetic foot”, “Foot, Diabetic”, “Diabetic Feet”, “Feet, Diabetic”, “Foot Ulcer, Diabetic”, “osteomyelitis”, “Osteomyelitides”, “rifampin”, “Benemycin”,“Rifampicin”, “Rimactan”, “Tubocin”, “Rifadin”, “Rimactane”. We will simply present the search process of the pubmed, as shown in Table 1.

**Table 1:**
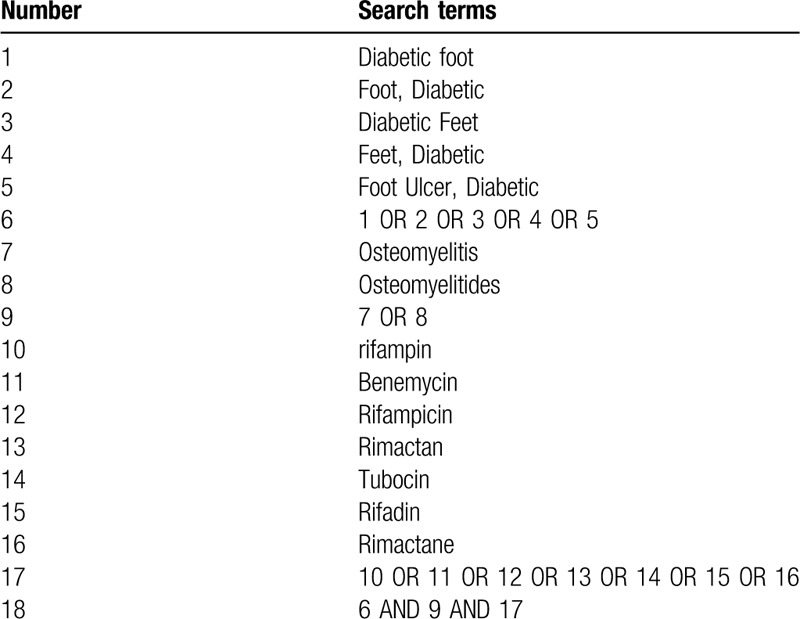
PubMed Medline search strategy.

### 2.6 Data extraction and management

According to the characteristics of this study, before extracting data, we prepared Excle for data extraction. Outcome indicators for eligible studies were independently extracted and filled in the data extraction form by 2 reviewers. If there is any objection during the data extraction process, we will be resolved by 2 reviewers’ discussions or by seeking comments from third reviewer. For each study, For each study, we will extract the following data, article title, author, year of publication, sample size, age, gender, course of disease, intervention measures, outcome indicators, adverse reactions, and so on. If there is not enough data in a study, we will contact the corresponding author for more detailed data. If the method details are not stated in the paper, we will contact more explanations.

### 2.7 Risk of bias assessment

Each randomized controlled trial included requires an assessment of its risk of bias,^[27]^ and the 2 reviewers will use the Cochrane Collaboration tool to assess the risk of bias in the included article. This is a more reliable tool for assessing the risk of research bias. There are 7 items to assess the risk of bias in the trial: random sequence generation (selection bias), allocation of hidden (selection bias), participant and human blindness (performance bias), outcome evaluation blindness (detection bias), incomplete outcome data (Loss bias), selective reporting (reporting bias), and other biases. Each project is classified as “low risk”, “high risk” or “unclear risk”. The risk of bias will be assessed independently by 2 reviewers and any differences will be resolved through discussion by all reviewers.

### 2.8 Statistical analysis

We will perform a meta-analysis of all statistics using Revman 5.3 software provided by Cochrane Collaboration, use 95% confidence intervals and risk ratio to calculate categorical variables, and use 95% confidence intervals and mean difference Calculate continuous variables. If there is no heterogeneity in the trial, study design of participants, controls, interventions, and outcome measures (*I*^2^ < 50%, *P* > .1), the data are synthesized using a fixed effects model. Otherwise (*I*^2^ ≥50%, *P* < .1), a random effects model will be used for analysis. Finally, subgroup analysis was performed based on different causes of heterogeneity, and if meta-analysis was not available, it was replaced with general descriptive analysis.

### 2.9 Subgroup analysis

If the results of the study are heterogeneous, we will conduct a subgroup analysis for different reasons. Heterogeneity is manifested in the following several aspects, such as race, age, gender, different intervention forms, pharmaceutical dosage, treatment course.

### 2.10 Sensitivity analysis

In order to study the stability of the results, we will conduct a sensitivity analysis of the results. Exclude each study included in the analysis 1 by 1, re-analyze and summarize the data, and compare the differences between the retrieved results and the original results. Therefore, we will be able to discover the impact of individual studies on overall results and whether the results are reliable.

### 2.11 Publication bias assessment

If there are more than ten studies in this meta-analysis, the publication bias will be reported through the funnel plot and the Egger test. For the Egger test, if *P* < .05, the publication bias will be considered.

### 2.12 Grading the quality of evidence

The quality of evidence in this study was evaluated using “Recommendation Evaluation, Development and Evaluation System Level (GRADE)”, a widely used tool for quality assessment, established by the World Health Organization and international organizations.^[28]^ To make the evidence simple and transparent, the system divides the quality of the evidence into 4 levels: high, medium, low, and very low. This systematic review will be analyzed using GRADE profiler 3.2.

### 2.13 Ethics and dissemination

This systematic review does not require approval from the ethics committee. This study systematically evaluated the existing research evidence of rifampicin adjuvant therapy DFO, which will certainly provide evidence-based medicine support for clinical workers. Our findings will be published in a peer-reviewed journal.

## 3 Discussion

DFO is a serious and complicated complication of diabetes, which is difficult to diagnose and treat. The goal of DFO treatment is to preserve as much normal foot tissue as possible, maintain foot stability and restore its function, and avoid recurrence of ulcers and amputations. At the same time, blood glucose, blood pressure, blood lipids and body weight should be controlled. The nerve and blood vessels of lower limbs should be paid close attention to whether there are damage, and pay attention to the care of the feet. If the injury is found, it should be dealt with in time to prevent the deterioration of the disease, reduce the amputation rate and improve the quality of life of patients with diabetes.

At present, it is still controversial which method is better for treating DFO. The choice of antibiotics and the course of treatment vary from person to person. There are no definite guidelines for clinical treatment of DFO. However, the more active use of antibiotics can reduce the amputation level. Rifampicin's broad antibacterial spectrum, strong bactericidal activity, tissue penetration, and biofilm activity have achieved good results not only in clinical trials of non-diabetic osteomyelitis, but also played an active role in the adjuvant treatment of DFO, reducing the amputation rate and mortality of patients.^[9,22,23]^ However, there is currently no systematic summary of clinical evidence, which limits the clinical application of rifampicin. Therefore, we attempted to perform this meta-analysis to provide high-quality evidence for the clinical efficacy and safety of rifampicin in the adjuvant treatment of DFO. The purpose of this systematic review and meta-analysis will to evaluate the effect of rifampicin in the treatment of DFO on amputation rates and mortality in patients. This study can not only provide a theoretical basis for the prevention and treatment of DFO, promote the application of rifampicin and benefit more patients in the future.

## Author contributions

**Conceptualization:** Yanli Zhang

**Data curation:** Yanli Zhang, Shengju Wang, Min Liu

**Formal analysis:** Shasha Yao, Song Fang

**Funding acquisition:** Qiu Chen

**Methodology:** Yanli Zhang, Min Liu, Song Fang

**Project administration:** Qiu Chen

**Resources:** Yanli Zhang, Min Liu, Haiping Cheng

**Software:** Yanli Zhang, Shengju Wang, Min Liu

**Supervision:** Qiu Chen

**Writing – original draft:** Yanli Zhang, Shengju Wang

**Writing – review and editing:** Qiu Chen
